# A longitudinal study of suicide and suicide attempt in northwest of Iran: incidence, predictors, and socioeconomic status and the role of sociocultural status

**DOI:** 10.1186/s12889-021-11527-9

**Published:** 2021-07-30

**Authors:** Ali Fakhari, Mostafa Farahbakhsh, Elham Davtalab Esmaeili, Hosein Azizi

**Affiliations:** 1 Research Center of Psychiatry and Behavioral Sciences, Tabriz University of Medical Sciences, Tabriz, Iran; 2 Medical Education Research Center, Tabriz University of Medical Sciences, Tabriz, Iran; 3 Road Traffic Injury Research Center, Tabriz University of Medical Sciences, Tabriz, Iran; 4 Social Determinants of Health Research Center, Health Management and Safety Promotion Research Institute, Tabriz University of Medical Sciences, Tabriz, Iran; 5 Department of Epidemiology and Biostatistics, School of Public Health, Tehran University of Medical Sciences, Tehran, Iran

**Keywords:** Incidence, Iran, Sociocultural status, Socioeconomic status, Suicide attempt

## Abstract

**Background:**

A detailed community-level understanding of socioeconomic status (SES) and sociocultural status (SCS) of suicides and suicide attempters (SAs) in a prospective design could have significant implications for policymakers at the local prevention and treatment levels. The effect of SCS and SES on SAs is poorly understood and investigated in Iran. The present study aimed to investigate the incidence, trend, and role of SES and SCS on suicide and SAs.

**Methods:**

A longitudinal study was conducted based on the registry for SAs in Malekan County, Iran, from 2015 to 2018. Demographic characteristics, SES, SCS, incidence rates, and predictors of suicidal behaviors were measured via structured instruments. Simple and multiple logistic regressions were used to estimate crude and adjusted odds ratios (ORs) and 95% confidence intervals (CIs).

**Results:**

A total of 853 SAs (32 suicides and 821 attempts) were identified during the study. Trend analysis revealed that the suicide rate significantly decreased from 2014 (10.28) to 2018 (1.75) per 100,000. In the final multiple variable models, age (26–40), male sex, unemployment, antisocial activities, history of SA, hanging method, and season (spring) increased the suicide risk while religious commitment had protective effects on suicide.

**Conclusions:**

Our findings indicated that demographic characteristics, low SES, and SCS are associated with suicide. In this county, trend of suicide and SA were decreased from 2014 to 2018. This study findings highlight the need to consider a wide range of contextual variables, socio-demographic, SES, and SCS in suicide prevention strategies. Improving inter-sectoral collaborations and policymakers’ attitudes are imperative for SA reduction.

## Background

Suicide has been defined as death caused by self-directed injurious behavior with any intent to die due to this behavior. A suicide attempt (SA) is a nonfatal self-directed, potentially injurious behavior with any intent to die due to the behavior [1]. Suicide is responsible for almost one million deaths every year. On average, 132 suicides occur per day; in other words, more than one person every 40 s [2]. Currently, suicide is a high-burden phenomenon throughout the lifespan. It is a global concern that imposes enormous costs on health care systems. Worldwide, the age-adjusted suicide rate is 10.5 per 100,000 persons. In both sexes of young people aged 15–29, suicide is the second leading cause of death after road traffic accidents. The majority of suicides occur in low- and middle-income countries. These numbers are the tip of the iceberg and are under-reported due to the lack of a registry for suicide, effective suicide surveillance, and cultural and social stigma [3, 4].

In Iran, a 20-year trend indicates increased suicidal deaths with an estimated average rate of 9.9 per 100,000 persons per year. It is estimated that 200 years of life lost (YLL) per 100,000 persons are attributed to suicidal behaviors (SBs) and self-inflicted violence in Iran [5, 6].

Suicide is a complex and multifactorial issue determined by several demographic and low socioeconomic status (SES), mental illness, sociocultural status (SCS), history of SBs, and health systems’ performance concerning suicide prevention [5, 7]. SES indicators, such as educational level, unemployment, and low income, have been reliably recognized as risk factors for suicide and SB [8].

Prior to this study, a health community assessment indicated that SBs are the most important health priorities in Malekan County. The Primary Health Care (PHC) system of Malekan County implemented a suicide prevention program to reduce suicide and SBs and re-attempts during 2014–2018 [9]. The County suicide prevention program included six aspects: 1) establishing a research team, 2) improving registry for suicidal behaviors, 3) identifying local determinants of SBs, 4) training health care providers, 5) follow-up monitoring of SBs, (suicide and depression risk assessment to prevent re-attempt), and 6) public awareness campaigns.

A detailed community-level understanding of SES, SCS, and predictors of SBs in a prospective design could have significant implications for policymakers and health system managers at the local prevention and treatment level, especially the effect of SCS and SES on SBs that are poorly understood and investigated in Iran [10]. Likewise, demographic status in a particular age and sex is a significant determinant of suicide and SA. Suicide incidence rates and distributions are varied in terms of age, sex, methods, and seasons in different communities. Understanding these changes in key determinants of suicide can be effective in developing and implementing suicide prevention programs [11].

Suicide and SBs are affected strongly by SCS compared to other diseases. Culture might provide a support system for an individual’s vulnerability and defenses related to ego-functioning; on the other hand, it might perpetuate an ecologically unhealthy environment. Cultural factors cannot be ignored as they significantly correlate with the global incidence of death by suicide [12].

The main objective of this temporal study was to identify the incidence rate, predictors, and SES of SBs in Malekan County, Iran. Another aim was to determine the role of SCS in SBs based on the registry for SBs from 2015 to 2018.

## Methods

### Study design and setting

This study was conducted and extracted from a community-based suicide prevention and follow-up program [13] (during 2014–2018) in Malekan County throughout 2015–2018. This community-based suicide prevention project conducted many interventions against suicide and SBs and followed up suicide attempters for re-attempt prevention and identifying local risk factors in this community.

The present longitudinal (temporal) study (as a part of this project), the incidence rate, SES, SCS, and the main risk factors of suicides and SAs were measured in Malekan County from 2015 to 2018.

The study population and participants (sampling frame) included all SAs, and suicides, and the involved individuals in the County during 2015–2018 and registered in the registry for suicide system. The study samples were included all 821 SAs and 32 suicide cases of the native population in Malekan County during 2015–2018. This study involved also SAs and suicide cases of Malekan County who registered in the neighborhood areas or counties. Malekan County is located in northwestern Iran with a population of 111,319 people (female: 53,653; male: 57,666) according to the 2015 national census. The native language of all the people of this County is Turkish, and all of them are Muslims. Almost 70% of the County population lives in rural areas. Their main occupation is farming or farming-related [10].

In this study, SBs were collected via both hospital (emergency ward) and non-hospital sources (community health centers) Community Health Workers (CHWs) who have face-to-face contact with large numbers of community members as part of their routine performance [14], and health systems of neighboring cities and then registered in the registry system.

All SAs were followed up and interviewed after SA during the study period 2015–2018. The basic and primary information included name, age, sex, telephone number, residence, and attempted method collected by the emergency ward or community health centers. Attempts were made to increase the coverage of suicide and SA registry in the county, including 1) fast-collecting of SAs from the emergency ward by a simple and rapid checklist, 2) collecting lists of SAs referred to neighboring counties, such as Miyandowab, Bonab, and Maragheh, and 3) using native CHWs (*Behvarz* in Persian) to obtain valid information. CHWs created a medical file for those attempting suicide in the community health center to record demographic and healthcare services and invite and coordinate interviews with participants.

### Measurements

This study followed up and interviewed all SAs to prevent re-attempt and recognize risk factors on suicide and SA. A trained expert team, including psychologists and mental health experts, was used through face-to-face and structured interviews.

One to 2 weeks after each SA incident, we interviewed the participants face to face to measure demographic, SES, SCS, and risk factors of the participants by trained psychologists. Interviews were obtained in a single sitting and confident situation that lasted 45–60 min. Interviews by psychologists provide more information about many valid and actual aspects of SCS, SES, and psychological aspects of SAs. We interviewed the closest friend or family members, such as parents, spouses, and siblings of death cases (suicides), to obtain valid and reliable information. Furthermore, we used CHWs, who had face-to-face contact with large numbers of community members, to evaluate the reliability of previous interviews with the closest member and accurate information about suicide cases.

### Demographic characteristics

Demographic variables included sex, age, marital status, residence, living alone, and family size. Age was measured continuously and grouped as 10–25, 26–40, and ≥ 41 years. Marital status was measured as single, married/cohabiting, widowed and/or divorced.

### Socioeconomic status (SES)

SES was measured by a valid and reliable questionnaire previously developed and validated among Iranian samples in this province [15]. Socioeconomic indicators included educational level, family (household) income, and occupation/employment status. Gross monthly household income from all sources was categorically measured based on Iranian currency (Rials): less than 10, 10–20, 20–50, > 50 million Rials. Educational level was measured based on educational years and grouped as primary school, secondary school, high school, and academic. Occupation/employment status was measured by an open question in the interviews and categorized on students of the school and college (as a frequent group of SA in this County), housewife, farming or farming-related (a typical job in this area), self-employed, and unemployed.

### Trend and pattern of suicidal behaviors

The trend of SAs included incidence rates of suicide and SA by year, sex, and season. Incidence rates were measured via the number of SAs occurring based on registry system × 100,000/the number of County population in the specific time/year. Methods of SA, seasons, place, and the history of attempts were measured via the registry system and face-to-face interviews.

### Sociocultural status (SCS)

A valid social participation questionnaire among Iranian samples was used for measuring SCS. This questionnaire (Persian version) was used and validated by Rashedi et al. [16] and Darvishpoor et al. [17] in Iran. It uses a 4-point Likert-type scale (very minimum (never or poor) = 1, minimum (at least once a year) = 2, moderate (at least once a month) = 3, much (at least once a week) = 4).

SCS, as an individual social characteristic and participation, included “social/teamwork collaborations and/or participation,” “faith and/or religious commitment,” “individual assistance and helpfulness,” and “conflict/antisocial status” [18].

Social/teamwork collaborations were defined as the strength of constructive relationships within groups in developing a common goal and/or membership in cultural and social associations in the community. Faith and religious commitment were defined as performing the religious duties and obligations of Muslims, including prayers, fasting, enjoining the good, forbidding the evil, etc. Individual assistance and helpfulness character was defined as a person who provides service to the people (materially and spiritually) and shares their grief. Also, conflict/antisocial status was defined as a long-term pattern of manipulating, exploiting, or violating the rights of others without any remorse [19, 20].

### Data analysis

The SPSS 21.0 (Chicago, IL, USA) was used for data analysis. Kolmogorov-Smirnov test was used to check data normality. Descriptive statistics, including charts and figures, were used to display SBs distribution by sex and seasons. Chi-squared (χ2) test was used to assess the relationship between SB and dichotomous variables. Simple and multiple logistic regressions were used to estimate the crude and adjusted odds ratios (ORs) with 95% confidence intervals (CIs) for the risk of suicide in the presence of variables. In all the tests, the confidence interval was considered 95%, and *P*-value< 0.05 was considered significant.

## Results

Table 1 shows the demographic status of SAs in Malekan County from 2015 to 2018. A total of 853 individuals with SBs were identified during the study period. Of this, 32 suicides and 821 SAs had been recorded in the registry for suicide. The majority of suicides were found in males with 23 cases (71.9%), while there were 525 SA cases (63.95%) in females. There was a positive, significant association between the male sex and the risk of suicide (*P* = 0.001). Moreover, the 26–40 age group had the highest frequency of suicide and SAs, and a significant association was found between age groups and suicide (*P* = 0.001). Regarding another demographic status, family size, living alone, and marital status were not associated with suicide risk (*P* > 0.05).

**Table 1:** Demographic status of suicidal behaviors in the Malekan County from 2015 to 2018

Variables	Suicidal Behaviors (853)		OR (95% CI)	***P***-value
	Attempters (***N*** = 821)	Suicides (***N*** = 32)		
Sex				
Female	525 (63.95)	9 (28.12)	1	1
Male	296 (36.05)	23 (71.9)	4.6 (1.99–10.63)	0.001
Age				
10–25	504 (61.39)	9 (28.125)	1	1
26–40	241 (29.35)	18 (56.25)	4.22 (1.75–10.15)	0.001
≥ 40	76 (9.25)	5 (15.63)	3.76 (1.1–12.92)	0.035
Marital status				
Single	140 (17.05)	10 (31.25)	1	1
Married	611 (74.42)	21 (65.63)	0.47 (0.20–1.12)	0.091
Widow and Divorced	70 (8.53)	1 (3.12)	0.2 (0.23–1.73)	0.144
Family size				
≥2	155 (18.83)	4 (12.5)	1.37 (0.72–3.27)	0.317
3–4	443 (53.9)	16 (50.00)		
≥4	223 (27.16)	12 (37.5)		
Life alone	43 (5.23)	1 (3.12)	0.94 (0.91–1.19)	0.530

**Fig. 1:**
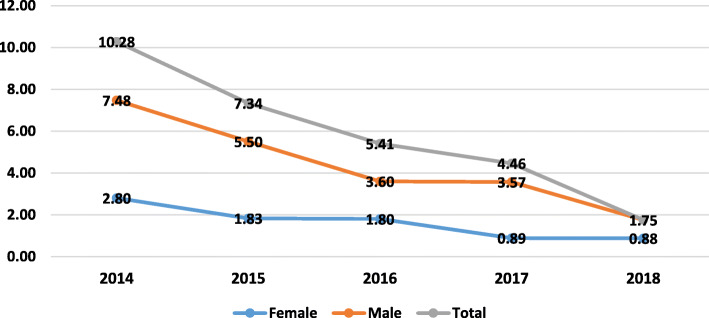
Incidence rates (per 100,000) of suicide (death) by sex in Malekan County (*r* = − 0.92, *P* = 0.001)

**Fig. 2:**
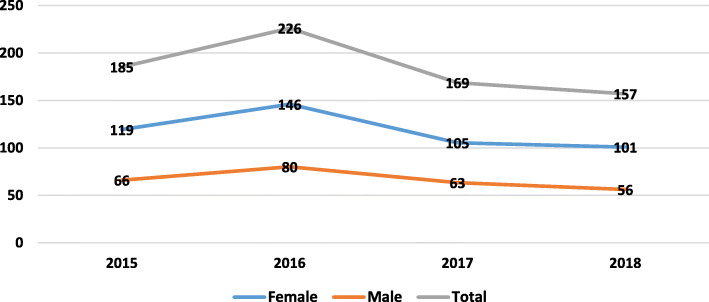
Incidence rates of suicide attempt (per 100,000) by sex in Malekan County from 2015 to 2018

Fig. 1 shows the trend of incidence rates of suicide by sex in Malekan County from 2014 to 2018. Trend analysis revealed that the suicide rate significantly decreased from 2014 to 2018 (*r* = − 0.92, *P* = 0.001). Likewise, the trend of the SA rate also significantly reduced during the study from 2014 to 2018. Moreover, suicide rates among males in all the years were higher than females. In contrast, SA rates among males in all the years were lower than females (*P* = 0.036), *r* = 0.903) (Fig. 2).

Table 2 presents the SES of SAs in Malekan County from 2015 to 2018. There was a positive relationship between low SES and suicide risk. Low educational level, occupation (unemployment), and rural area increased the risk of death from suicide significantly (*P* < 0.05). Furthermore, the findings revealed that high family income was a risk factor for successful suicide (*P* = 0.001). Moreover, some behavior characteristics, including alcohol, substance abuse, and current smoking, were associated with suicide risk (P < 0.05).

**Table 2:** Socioeconomic status (SES) of suicidal behaviors in the Malekan County from 2015 to 2018

Variables	Suicidal behaviors (***N*** = 853)		OR (95% CI)	***P***-value
	Attempters ***N*** = 821	Suicides ***N*** = 32		
Occupation				
Student (school/college)	181 (22.05)	7 (21.87)	1	1
Farming-related	24 (2.92)	2 (6.25)	2.42 (0.36–16.03)	0.357
Housewife	540 (66.77)	5 (15.63)	0.23 (0.07–0.80)	0.021
Self-employed and/or unemployed	76 (9.25)	18 (56.25)	1.43 (1.12–3.17)	0.038
Educational level				
Primary school	277 (33.74)	10 (31.25)	1.71 (0.72–2.82)	0.065
Secondary school	427 (52.00)	19 (59.38)	1.53 (1.13–4.21)	0.032
High school and Academic	118 (14.37)	3 (9.37)	1	1
Family income (million Rials)				
10>	383 (46.65)	8 (25.00)	1	1
10–20	304 (37.02)	13 (40.62)	2.05 (0.79–5.3)	0.138
20–25	91 (11.08)	4 (12.5)	2.11 (0.56–7.88)	0.263
25<	43 (5.24)	7 (21.88)	7.87 (2.24–27.57)	0.001
Resident				
Urban	166 (20.22)	2 (6.25)	1	1
Rural	655 (79.78)	30 (93.75)	3.22 (0.812–12.78)	0.079
Substance abuse	45 (5.48)	2 (6.250	1.11 (1.00–1.27)	0.042
Alcohol abuse	41 (4.99)	11 (34.37)	1.43 (1.12–1.85)	0.001
Current smoker	147 (17.90)	3 (9.37)	1.49 (1.12–2.06)	0.001

Table 3 shows the selected characteristics of individuals with SAs. Hanging (62%) was a frequent suicide method, whereas poisoning (75%) was a prevalent method among SAs, particularly in females. Therefore, the hanging method strongly increased suicide risk (OR: 8.5, 95% CI: 2.9–76.99). The history of SA significantly increased the suicide risk (*P* = 0.028). Regarding the place of SAs, the majority of both suicide (26, 81.25%) and SAs (747, 90.98%) had occurred in residential buildings.

**Table 3:** Selected characteristics of suicidal behaviors in Malekan County from 2015 to 2018

Variable	Suicide ***N*** = 32	Attempters ***N*** = 821	OR (95% CI)	***P***-value
Methods				
Hanging	20 (62.5)	11 (1.34)	8.5 (2.9–76.99)	0.001
Poisoning	8 (25)	613 (74.66)	0.63 (0.18–2.24)	0.483
Self-injury	2 (6.25)	159 (19.37)	0.446 (0.12–1.78)	0.236
Self-burning	2 (6.25)	38 (4.63)	1	1
History of SB				
Yes	8 (25.0)	47 (5.72)	2.59 (1.086–6.17)	0.028
No	24 (75.0)	774 (94.28)	1	1
Place				
Residential building or at home	26 (81.25)	747 (90.98)	2.23 (1.18–4.21)	0.013
Non-residential building	1 (3.125)	21 (2.55)		
Outdoor	5 (15.62)	53 (6.45)		

**Fig. 3:**
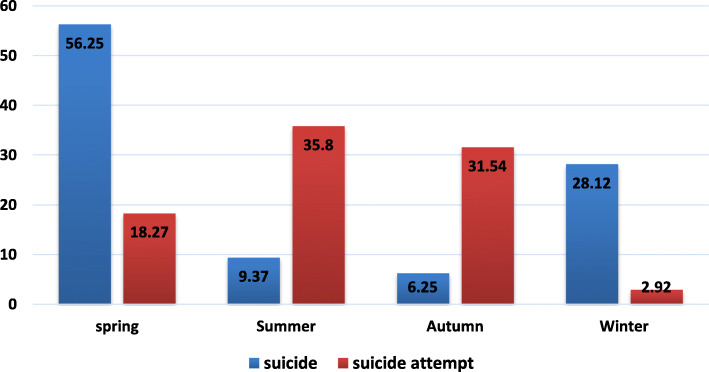
Prevalence rates of suicide and suicide attempt in terms of seasons in Malekan County from 2015 to 2018 (*P* = 0.001)

Fig. 3 shows the distribution of SAs by seasons from 2015 to 2018. Spring (18, 56.25%) was a frequent season for suicide, while summer (288, 35.8%) was a prevalent season for SAs. There was a positive association between spring and suicide risk (*P* = 0.001).

Table 4 shows the results of multiple logistic regression analysis and estimation of adjusted odds ratios (ORs) and 95% confidence intervals (CIs) for the suicide risk. Adjusting for the potential confounders showed that age (26–40), male sex, low educational level (non-academic), low occupation status (self-employment and/or unemployment), income (> 5 million Rials per monthly) were associated with the risk of suicide. Likewise, history of SA (AOR = 2.23, 95% CI: 1.70–6.45), hanging method (AOR = 12.62, 95% CI: 3.14–28.02), and season (spring) (AOR = 3.56, 95% CI: 2.19–9.63) increased the suicide risk.

**Table 4:** Results of multiple logistic regression to estimate the measure of associations and 95% confidence intervals for the risk of suicide

Variables	Adjusted OR (95% CI)	***P***- value
Age		
10–25	1	1
26–40	6.34 (2.1–19.15)	0.001
≥40	4.92 (0.8–30.58)	0.088
**Sex**		
Female	1	1
Male	3.48 (1.32–9.24)	0.012
**Income**		
≤10 million Rials	1	1
> 10 million Rials	2.68 (1.03–5.41)	0.049
**Educational level**		
Academic	1	1
Non-academic	2.24 (1.13–4.96)	0.027
**Occupation**		
Student (school or college)	1	1
Farming or farming-related	2.49 (0.138–45.05)	0.535
Housewife	0.198 (0.045–0.86)	0.032
Self-employed or unemployed	6.88 (1.73–27.53)	0.006
**Hanging method**	12.62 (3.14–28.02)	0.001
**History of suicidal behavior**	2.23 (1.70–6.45)	0.002
**Spring season**	3.56 (2.19–9.63)	0.001

Table 5 presents the results of simple and multiple logistic regression analysis and measure of associations of SCS on SAs among study participants. The results showed that having faith and religious commitment was a protective factor in suicide prevention in this study (AOR = 0.30, 95% CI: 0.17–0.53; *P* = 0.001). Likewise, having social and teamwork activities and a state of helpfulness and assistance were associated with suicide and SAs prevention. However, there was no significant evidence in the final analysis that these reduced the suicide risk (*P* > 0.05). Moreover, our results demonstrated a positive association between antisocial activities and the suicide risk (AOR = 1.37, 95% CI: 1.03–2.05; *P* = 0.048).

**Table 5:** Sociocultural status of suicides and suicide attempters in Malekan County from 2015 to 2018

Variables	Suicidal Behaviors (***N*** = 853)		Crude OR (95% CI)	Adjusted OR (95% CI)
	Attempters (***N*** = 821)	Suicides (***N*** = 32)		
Social and teamwork activities				
Very minimum	90 (11.0)	7 (21.9)	0.89 (0.61–1.30)	1.18 (0.77–1.81)
Minimum	171 (20.8)	1 (3.1)		
Moderate	293 (35.7)	17 (56.3)		
Much	267 (32.5)	7 (21.9)		
***P*-value**			0.515	0.444
**Religious commitment**				
Very minimum	0 (0.0)	3 (9.4)	0.33 (0.20–0.56)	0.30 (0.17–0.53)
Minimum	229 (27.9)	16 (50.0)		
Moderate	224 (27.3)	10 (31.3)		
Much	368 (44.8)	3 (9.4)		
***P*-value**			0.001	0.001
**Helpfulness and assistance**				
Very minimum	496 (60.4)	18 (56.3)	0.86 (0.57–1.29)	0.75 (0.47–1.22)
Minimum	154 (18.8)	12 (37.5)		
Moderate	101 (12.3)	2 (6.3)		
Much	70 (8.4)	0 (0.0)		
***P*-value**			0.467	0.244
**Antisocial activities**				
Very minimum	453 (55.2)	19 (59.4)	1.24 (0.89–1.73)	1.37 (1.03–2.05)
Minimum	203 (24.7)	2 (6.3)		
Moderate	85 (10.4)	4 (12.5)		
Much	80 (9.7)	7 (21.9)		
***P*-value**			0.181	0.048

## Discussion

This registry-based prospective study was conducted on individuals with SAs (suicide and SAs) in Malekan County from 2015 to 2018. Face-to-face interviews were performed to measure the role of SCS, SES, demographic status, and trend of incidence rates of suicide and SA in terms of sex and the main predictors of suicide. The study outcome refers to whole community (population) of Malekan County, but not for a group of individuals of community.

In this community-based and health system research study, suicide and SA data were collected from various hospital and non-hospital sources. We used the regional (Malekan County) health system data and resources, as well as the health system of neighborhood areas, and community healthcare providers to achieve valid findings in a prospective design with psychological interviews and followed up of attempters.

Since suicide cases are similar to the tip of the iceberg, community-based methods and efforts that used for collecting suicide and SA statistics and risk factors in this study can present a valid suicide and suicidal behaviors situation in this area and Iranian context. Moreover, the finding of this study highlights the importance role of this study and suicide research, and prevention strategies for policymakers and health managers by considering that the incidence rate of suicide has increased in Iran over the past decades which there are limited studies on suicide [21]. In this study, collecting and evaluating the impact of all the contextual variables, risk factors, socio-economic factors, and also SCS on suicide will help to better understand their effects on suicide risk or prevention.

Regarding the demographic characteristics and SES, the final analysis showed that male sex, the age range of 26–40, low educational level, low occupational status (unemployed or self-employed), income (> 10 million Rials), and rural area were risk factors for suicide. Aschan et al. in South East London found that low SES was directly associated with SAs [8]. Consistent with our findings, a systematic review revealed that low SES, low educational level, and low occupational status were related to suicide risk [22].

In this study, suicide deaths were prevalent in high-income households, while SA was prevalent in low-income households. A review study found that low economic status, weakened wealth, and unemployment were associated with SBs, but they reported insufficient data to draw clear conclusions at the country level [23].

Our trend analysis showed that the incidence of suicide and SA decreased in the County from 2015 to 2018. Also, in all the years, the incidence of suicide in males was higher than in females, while SA had quite the opposite situation compared to suicide. The reason for the decrease in suicide and SA rates during the study was the implementation of a community-based suicide prevention program in the County health system from 2014 to 2018. However, long term period is needed for evaluating the effectiveness of the interventions.

In this study, there was a protective relationship between faith and religious commitment and suicide, while a negative association was found between antisocial activities and suicide risk. Consistent with our results, a study from 25 nations found a negative association between religious commitment and suicide rates [24]. Besides, a study reported that the acceptability of suicide is lower among people in nations with relatively high levels of religiosity, who are affiliated with one of the four major faiths, are religiously committed, and are engaged with a religious network [25]. The same results were reported by Malaysian [26]. Suicide and SBs rates were lower in Islamic countries [5], indicating that affiliation with Islam was associated with low suicide acceptability [27, 28]. However, a recent study reported the relevance of individual characteristics rather than a worldwide pattern and questions the importance of religion [29].

However, there was no significant association between individuals with teamwork activities and helpfulness or assistance characteristics and suicide prevention in the present study. However, in the present study, the teamwork and helpfulness aspects as individual social characteristics were poorly associated with suicide prevention; nevertheless, there was no decrease in successful suicide in the final analysis. Nonetheless, comparing these aspects with controls from the general population might reveal additional information. Consistent with our findings, Martikianen et al. and a study in Denmark did not find a significant relationship between individual social characteristics and suicide mortality [30, 31].

Another finding of this study was that suicidal individuals mostly selected the hanging method for death, so that a very strong association was found between the hanging method and the risk of suicide. Consistent with the present study, Kolves et al. analyzed 8140 suicides and found a significant increase in hanging in both sexes and poisoning with drugs in females [32]. A study in India showed that suicide by hanging increased by 56 and 24% among males and females, respectively [33]. It seems that the hanging method is an aggressive method that increases the odds of successful suicide. Therefore, restriction of access to suicide means by the regional health system could be an important strategy in suicide prevention. The present study showed that the hanging method was prevalent among suicides while the poisoning method, especially with medical drugs, was prevalent among SAs. Currently, a study in Iran confirmed that drug abuse and poisoning were the most common methods of SA; furthermore, demographic characteristics were the most important factors in predicting suicide death [34].

Most studies from both the Northern and the Southern hemispheres have confirmed a seasonal pattern and variation for suicide [35]. The present study showed the spring increased the risk of suicide. In the present study, the majority of suicides occurred in the spring. A review study by Christodoulou et al. confirmed a peak in spring, mainly for men, older individuals, and violent methods of suicide [35]. The seasonality of suicide was also confirmed in other Iranian studies, indicating that suicide peaks emerged in warm months [36, 37].

The present study showed the high prevalence of suicide mortality among non-academic, unemployed and/or self-employed individuals. Consistent with the present study, a study indicated that educational level, sex, and age were critical risk factors for suicide in Iran [38]. Similar results were found by a systematic review and meta-analysis for the association between suicide and occupation [39]. Another study found that construction workers and plant and machine operatives had the highest suicide numbers in England and Wales [40].

Moreover, the present study showed that committing suicide at home or residential buildings was more prevalent than in outdoor settings. Consistent with the findings of this study, Kposowa et al. reported that suicide is prevalent at home [41]. A study in Kentucky showed that the frequency and pattern of inside suicide were higher than outside pattern [42]. These different clusters of suicides, inside (at home) versus outdoor, can advise feasible and effective interventions and guide future researches.

To the best of our knowledge, this prospective study, unlike other studies that focused on a single subject or were individual-based, is one of the rare population- and register-based studies that measured various aspects of suicide and SA, including SCS, demographics, and SES, and the trend of suicide and SAs in a longitudinal design.

### Limitations

This prospective study revealed interesting and important results about SES, social and cultural aspects of SAs, and incidence rates of SAs based on a population registry-based system. However, there were some limitations. This study was a non-control prospective study and might do not allow for causal inferences. Comparison of suicides with controls without a history of SB can ascertain high strength of association. To diminish this problem, we used multiple logistic regression analyses to estimate the adjusted measures of associations and compared its findings with systematic reviews and population-based studies. On the other hand, death by suicide imposes a huge emotional and financial burden on health systems and communities. Our main objective was to identify predictors, trends, and prevention of suicide in the county, not SA, and the study achieved its objectives.

Another concern was several large 95% CIs due to the low sample size of suicide cases (*N* = 32) in comparison with SAs (*N* = 821) to explore associated risk factors. However, this study was used multiple logistic regression analysis to estimate the adjusted measure of associations (odds ratios) for suicide risk.

Moreover, the current study was performed in a small area with limited population. Therefore, the application of its methods and findings in large communities should be with caution.

## Conclusions

We found that demographic characteristics, low SES, and SCS have associated with suicide. In the final multiple variable model, age (26–40), male sex, low educational level, unemployment, antisocial activities, history of SA, hanging method, and season (spring) increased suicide risk while religious commitment and having social participation were associated against suicide. Furthermore, suicide and SA rates decreased from 2014 to 2018 in the county.

The practical framework that emerged in this research will provide a basis for developing an evidence-based suicide prevention strategies in Iranian and other contexts. This study findings challenge the personal-based risk-factor models of suicide prevention and highlight the need to consider a wide range of contextual, socio-demographic, and economic characteristics, and socio-cultural status (SCS) when developing and implementing strategies and programs against suicide and SA. Likewise, improving inter-sectoral collaborations and support from policymakers concerning SCS and SES are imperative for SA reduction.

## Data Availability

The datasets generated and/or analyzed during the current study are available from the corresponding author on reasonable request.

## Abbreviations

CIs: Confidence intervals
CHWs: Community health workers
PHC: Primary health care
ORs: Odds ratios
SES: Socioeconomic status
SCS: Sociocultural status
SAs: Suicide attempters
SBs: Suicidal behaviors
YLL: Years of life lost
